# MRI‐based radiomics nomogram to predict synchronous liver metastasis in primary rectal cancer patients

**DOI:** 10.1002/cam4.3185

**Published:** 2020-05-31

**Authors:** Minglu Liu, Xiaolu Ma, Fu Shen, Yuwei Xia, Yan Jia, Jianping Lu

**Affiliations:** ^1^ Department of Radiology Changhai Hospital Shanghai China; ^2^ Huiying Medical Technology Co., Ltd Beijing China

**Keywords:** magnetic resonance imaging, radiomics, rectal cancer, synchronous liver metastasis

## Abstract

At the time of diagnosis, approximately 15%‐20% of patients with rectal cancer (RC) presented synchronous liver metastasis (SLM), which is the most common cause of death in patients with RC. Therefore, preoperative, noninvasive, and accurate prediction of SLM is crucial for personalized treatment strategies. Recently, radiomics has been considered as an advanced image analysis method to evaluate the neoplastic heterogeneity with respect to diagnosis of the tumor and prediction of prognosis. In this study, a total of 1409 radiomics features were extracted for each volume of interest (VOI) from high‐resolution T2WI images of the primary RC. Subsequently, five optimal radiomics features were selected based on the training set using the least absolute shrinkage and selection operator (LASSO) method to construct the radiomics signature. In addition, radiomics signature combined with carcinoembryonic antigen (CEA) and carbohydrate antigen 19‐9 (CA19‐9) was included in the multifactor logistic regression to construct the nomogram model. It showed an optimal predictive performance in the validation set as compared to that in the radiomics model. The favorable calibration of the radiomics nomogram showed a nonsignificant Hosmer‐Lemeshow test statistic (*P* > .05). The decision curve analysis (DCA) showed that the radiomics nomogram is clinically superior to the radiomics model. Therefore, the nomogram amalgamating the radiomics signature and clinical risk factors serve as an effective quantitative approach to predict the SLM of primary RC.

## Graphical abstract

In the present study, we aimed to evaluate the predictive performance of radiomics nomogram for the diagnosis of synchronous liver metastasis in rectal cancer patients.

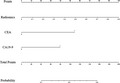

## 1 INTRODUCTION

Rectal cancer (RC) is one of the most commonly diagnosed malignant tumors worldwide. At the time of or before the diagnosis of the primary tumor, approximately 15%‐20% of the patients were detected with liver metastases (LM), which is termed as synchronous liver metastasis (SLM).^1^, ^2^ SLM is the most common cause of death in patients with RC,^3^ and the prognosis of patients with untreated SLM is poor.^4^ Therefore, the preoperative prediction of RC patients with a high risk of SLM is essential for treatment strategies. In the case of patients with high‐risk RC, further imaging, including enhanced abdomen computed tomography (CT), liver magnetic resonance imaging (MRI), or fluorodeoxyglucose (FDG) positron emission tomography (PET)‐CT, should be considered, to discover additional metastases, enhance the systemic treatment, or for metastasis resection.^5^ The early detection of SLM will provide opportunities for early intervention and improve the prognosis of RC patients.

Owing to high sensitivity and specificity over the other two modalities, MRI was the preferred first‐line approach for the preoperative clinical evaluation of SLM, especially for lesions smaller than 1 cm. PET‐CT is served as the second‐line approach.^5^ However, the diagnostic accuracies of these imaging techniques are not satisfactory.^5^, ^6^, ^7^ Previous studies demonstrated the feasibility of clinicopathological features for evaluating the potential risk of SLM in RC patients.^8^, ^9^ However, some indicators are available only after a radical resection and cannot be used as a guide for preoperative treatment strategy. Therefore, the development of a preoperative, noninvasive, and accurate approach is warranted to predict SLM.

Since the radiomics analysis of images provides comprehensive quantification information than that by a physician, the quantitative and objective descriptions of neoplastic heterogeneity could serve as alternatives in clinical studies. The radiomics workflow involves high‐throughput extraction of numerous medical imaging features. Then, the quantitative imaging traits are subjected to a selection procedure, for feature analysis, which supports the decision‐making^10^, ^11^, ^12^ with respect to the cancer stage and the prediction of prognosis.^13^, ^14^, ^15^ Another study demonstrated that the texture analysis of the features extracted from liver CT images predicted the different prognosis of colorectal cancer patients.^16^ Some studies demonstrated that radiomics model predicts distant metastasis in different primary tumors.^17^, ^18^, ^19^, ^20^, ^21^, ^22^ However, the role of radiomics nomogram derived from primary lesion in predicting SLM in RC patients is not yet clarified.

Therefore, the present study aimed to investigate the predictive performance of radiomics nomogram for the diagnosis of SLM in RC patients.

## 2 MATERIALS AND METHODS

### 2.1 Patients and data management

Research was approved by the local institutional review board (Committee on Ethics of Biomedicine, Naval Medical University, PLA), and patient informed consent was waived for this retrospective study. Between March 2018 and March 2019, a total of 169 patients with RC identified by histopathological examination were enrolled in this study. All subjects underwent rectal MRI for local staging of RC. The inclusion criteria included the following: (a) rectal adenocarcinoma was identified via colonoscopy or postoperative pathological examination; (b) single focus; (c) liver metastasis was confirmed by contrast‐enhanced liver MRI at the time of diagnosis. Exclusion criteria were as follows: (a) any local or systemic treatment at or before the baseline MRI examination (n = 21); (b) history of previous or coexisting other malignant tumors (n = 2); (c) metachronous liver metastasis (n = 11); (d) low‐quality image quality (n = 8). Therefore, the final study cohort consisted of 127 patients. The levels of serum tumor markers, carcinoembryonic antigen (CEA), and carbohydrate antigen 19‐9 (CA19‐9), were recorded consecutively to that of rectal MRI (the interval was <2 weeks). Next, the random number method was used to assign 70% of the samples to the training set and 30% to the validation set.

### 2.2 Imaging acquisition

All patients were performed on a 3.0 Tesla MRI scanner (MAGNETOM Skyra, Siemens Healthcare) with an 18‐channel phased‐array body coil. Rectal axial high‐resolution T2‐weighted fast spin echo images were obtained for subsequent processing as the following main scanning parameters: repetition time/echo time (TR/TE) = 4000/108 ms, field of view (FOV) = 180 × 180 mm^2^, matrix = 320 × 320, slice thickness = 3 mm, interspace = 0 mm, number of slices = 28 slices, echo train length = 16, without fat saturation and acquisition time = 4 minutes 10 seconds. Preoperative liver MR scan for liver metastases was acquired at an interval of 6.9 ± 1.8 (range, 2‐9) days before or after the rectal examination.

Different medical imaging factors cause inconsistencies in the image intensity information of tissues of the same nature. We used the following formula for intensity normalization (where *x* represents the original intensity; *f*(*x*) indicates the normalized intensity; *µ* indicates the average value; *σ* refers to variance; *t* is an optional scaling ratio, which has been set to 1 by default). While retaining the intensity difference of diagnostic value, the image intensity inconsistency caused by the difference in imaging parameters is reduced or even eliminated for subsequent imaging radiomics analysis.

$$f\left(x\right)=\frac{t(x-\mu_{x})}{\sigma_{x}}$$

### 2.3 Feature extraction and radiomics signature construction

**FIGURE 1:**
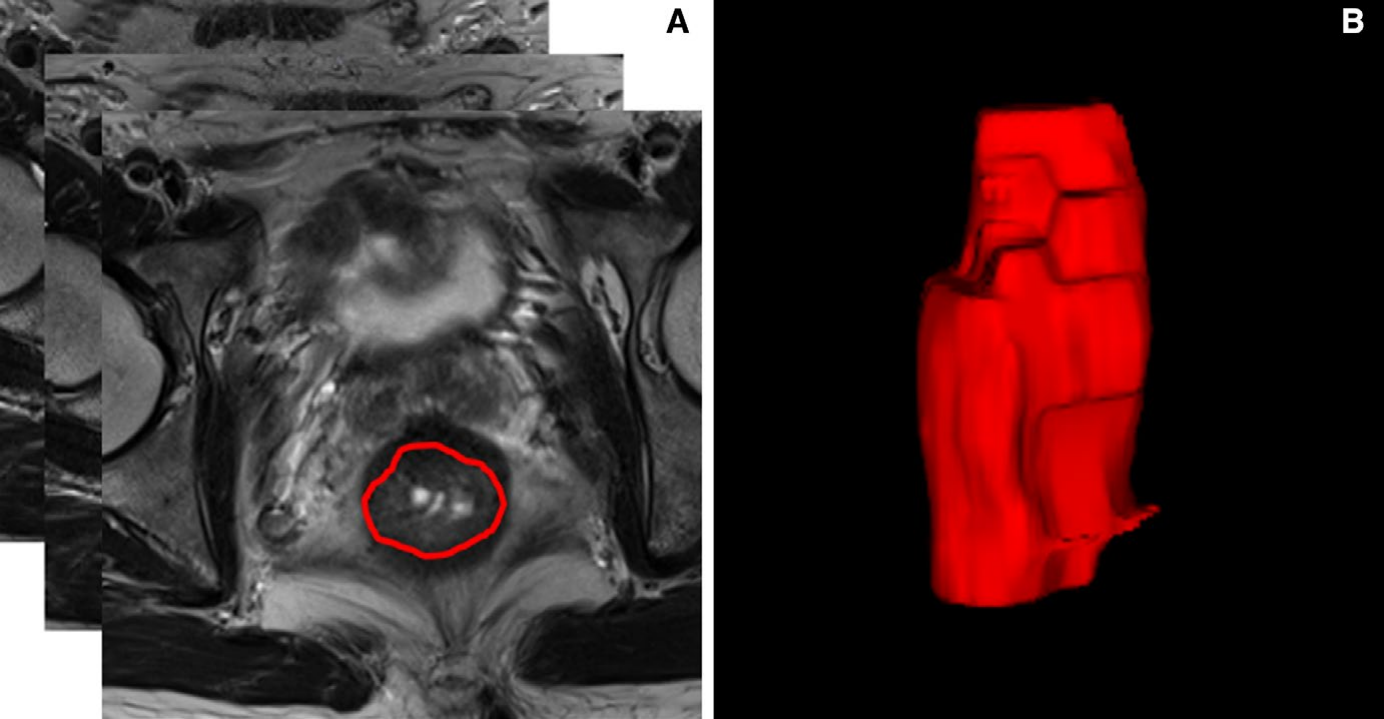
Example image for rectal cancer contouring. A, The outline of regions of interest on one slice of axial T2‐weighted MR image. B, Volume rendering

The regions of interest (ROIs) were manually delineated for all slices on high‐resolution T2WI based on radiomics analysis platform (Radcloud, Huiying Medical Technology Co., Ltd.) by one observer with 5 years of experience in radiology, followed by a review by one senior observer with >10 years’ experience. If the discrepancy was ≥5%, the senior observer decided on the ROI borders. Then, the volume of interest (VOI) was constructed through the ROI interpolation of each slice. A total of 127 VOIs were manually dropped from the scans of 127 patients (Figure 1). Subsequently, 1409 high‐throughput data features were automatically extracted from the platform based on the “pyradiomics” package in Python (version 2.1.2, https://pyradiomics.readthedocs.io/) and classified into four groups. (a) First‐order statistics (n = 18), described the intensity information in the MR image region of interest, such as mean, standard deviation, variance, maximum, median, range, etc (b) Shape features (n = 14), which reflected the shape and size of the region, such as volume, surface area, compactness, maximum diameter, etc (c) Texture features, which could quantify regional heterogeneity differences, such as gray‐level co‐occurrence matrix (GLCM, n = 24), gray‐level size zone matrix (GLSZM, n = 16), gray‐level dependence matrix (GLDM, n = 14), neighborhood gray‐level dependence matrix (NGLDM, n = 5), and gray‐level run‐length matrix (GLRLM, n = 16). (d) higher‐order statistical features, contained 1302 features, including the first‐order statistics and texture features derived from wavelet transformation of the original images: logarithm, exponential, gradient, square, square root, local binary patterns (LBP), the wavelet transform decomposes the tumor area image into low‐frequency components (L) or high‐frequency components (H) in the three directions of the *x*, *y*, and *z* axes. Eight types of wavelet features were obtained and labeled as LLL, LLH, LHL, LHH, HLL, HLH, HHL, and HHH according to their different decomposition orders.

First, we applied a variance threshold method to reduce the features, with the threshold setted to 0.8. On this basis, the least absolute shrinkage and selection operator (LASSO) method was used to screen the optimal features to predict the SLM. In this method, leave‐one‐out cross‐validation was used to select the optimal regularization parameter alpha, as the average of mean square error of each patient was small. Moreover, features with non‐zero coefficient in LASSO were utilized to establish the radiomics signature and calculate the score.

### 2.4 Prediction model of the radiomics nomogram

Univariate logistic regression was applied to the training set for each potential predictor, including gender, age, primary RC location, MR T stage, MR N stage, mesorectal fascia (MRF), extramural vascular invasion (EMVI), CEA, and CA19‐9 to choose the independent clinical prediction indicator. Then, multivariable logistic regression combined the selected risk factors, and radiomics signature was performed to develop a prediction model for SLM, following which, the radiomics nomogram was constructed. The calibration curve and Hosmer‐Lemeshow test were applied to estimate the goodness of fit of the model. Moreover, receiver operator characteristic (ROC) curve was constructed to compare the prediction performance of the single radiomics signature model and radiomics nomogram by calculating the area under the curve (AUC) in both the training and validation sets. The decision curve analysis (DCA) was effectuated by estimating the net benefit with probability thresholds to confirm the clinical benefit.

### 2.5 Statistical analysis

Radcloud platform (Huiying Medical Technology Co., Ltd) was utilized to process the imaging and clinical data, as well as the spectra of radiomics analysis. The nomogram analysis was performed using R software (version 3.3.1). Other statistical analysis were performed using SPSS software (version 17.0) and MedCalc (version 15.2.2). A value of *P* < .05 was considered statistically significant.

## 3 RESULTS

### 3.1 Patient demographics

A total of 127 patients with RC were incorporated in the final analysis, including 32 cases of SLM. Subsequently, 88/127 (70%) were assigned to training sets, and then remaining to validation sets. Any significant difference was not detected between the two sets (Table 1).

**TABLE 1:** Patient demographics

Variables	Total	Training data (70%)	Validation data (30%)	Statistic	*P*
	n = 127 (%)	n = 88 (%)	n = 39 (%)		
Gender					
Male	90 (70.9)	63 (71.6)	27 (69.2)	0.073^a^	.787
Female	37 (29.1)	25 (28.4)	12 (30.8)		
Age (y)					
Mean ± SD	57.0 ± 10.6	57.8 ± 10.2	55.3 ± 11.2	‐1.257^b^	.211
Location					
Upper	26 (20.5)	17 (19.3)	9 (23.1)	0.273^a^	.872
Middle	82 (64.6)	58 (65.9)	24 (61.5)		
Lower	19 (14.9)	13 (14.8)	6 (15.4)		
mr T stage					
T1‐2	43 (33.9)	29 (33.0)	14 (35.9)	0.105^a^	.746
T3‐4	84 (66.1)	59 (67.0)	25 (64.1)		
mr N stage					
N0	85 (66.9)	57 (64.8)	28 (71.8)	0.602^a^	.438
N1‐2	42 (33.1)	31 (35.2)	11 (28.2)		
MRF					
Negative	103 (81.1)	72 (81.8)	31 (79.5)	0.096^a^	.757
Positive	24 (18.9)	16 (18.2)	8 (20.5)		
EMVI					
Negative	85 (66.9)	57 (64.8)	28 (71.8)	0.602^a^	.438
Positive	42 (33.1)	31 (35.2)	11 (28.2)		
CEA					
Negative	82 (64.6)	58 (65.9)	24 (61.5)	0.226^a^	.635
Positive	45 (35.4)	30 (34.1)	15 (38.5)		
CA19‐9					
Negative	101 (79.5)	69 (78.4)	32 (82.1)	0.220^a^	.639
Positive	26 (20.5)	19 (21.6)	7 (17.9)		

^a^ *χ* ^2^ – value.

^b^ *t* – value.

### 3.2 Radiomics features

**FIGURE 2:**
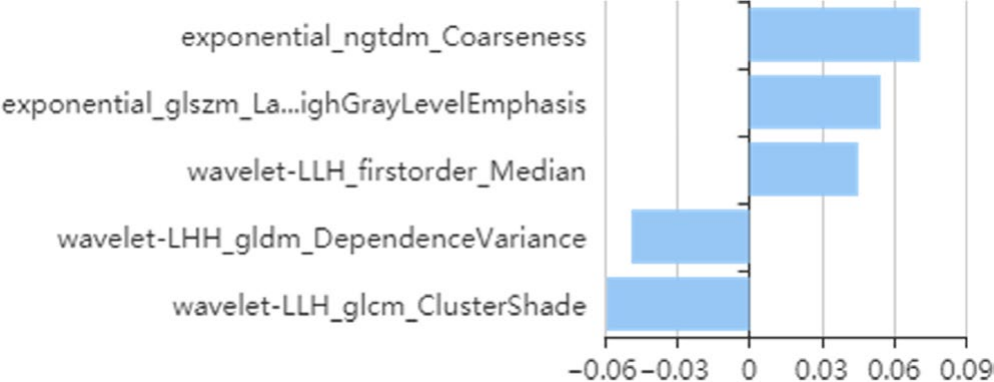
Radiomics features selected by least absolute shrinkage and selection operator (LASSO) algorithm. Lasso algorithm for feature selection. Five features were selected

First, we selected 866/1409 features using variance threshold method, and then, five optimal features were selected by LASSO algorithm (Figure 2). The radiomics score was obtained by a linear combination of optimal features that was respectively weighted by the LASSO coefficients that reflect the risk of SLM. While training with the radiomics signature, the AUC was 0.836 (95% confidence interval, CI: 70.64%‐96.50%), sensitivity was 100.0%, and specificity was 75.00% with a prediction accuracy of 81.58%. The AUC was 0.866 (95% CI: 76.96%‐96.32%), sensitivity was 79.17%, and specificity was 93.65% in the validation set, with an accuracy of 89.66%.

### 3.3 Radiomics nomogram

**FIGURE 3:**
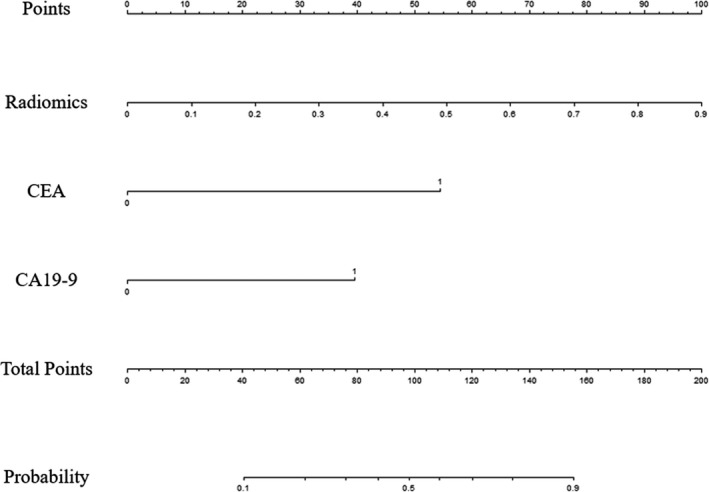
Radiomics nomogram to detect synchronous liver metastasis (SLM). The radiomics nomogram was developed in the training set with radiomics signature and tumor markers. In the nomogram, first, a vertical line was drawn according to the value of radiomics signature to determine the corresponding value of points. Similarly, the points of tumor markers were determined. The total points were the sum of the two points above. Finally, a vertical line was made according to the value of the total points to determine the probability of SLM

**FIGURE 4:**
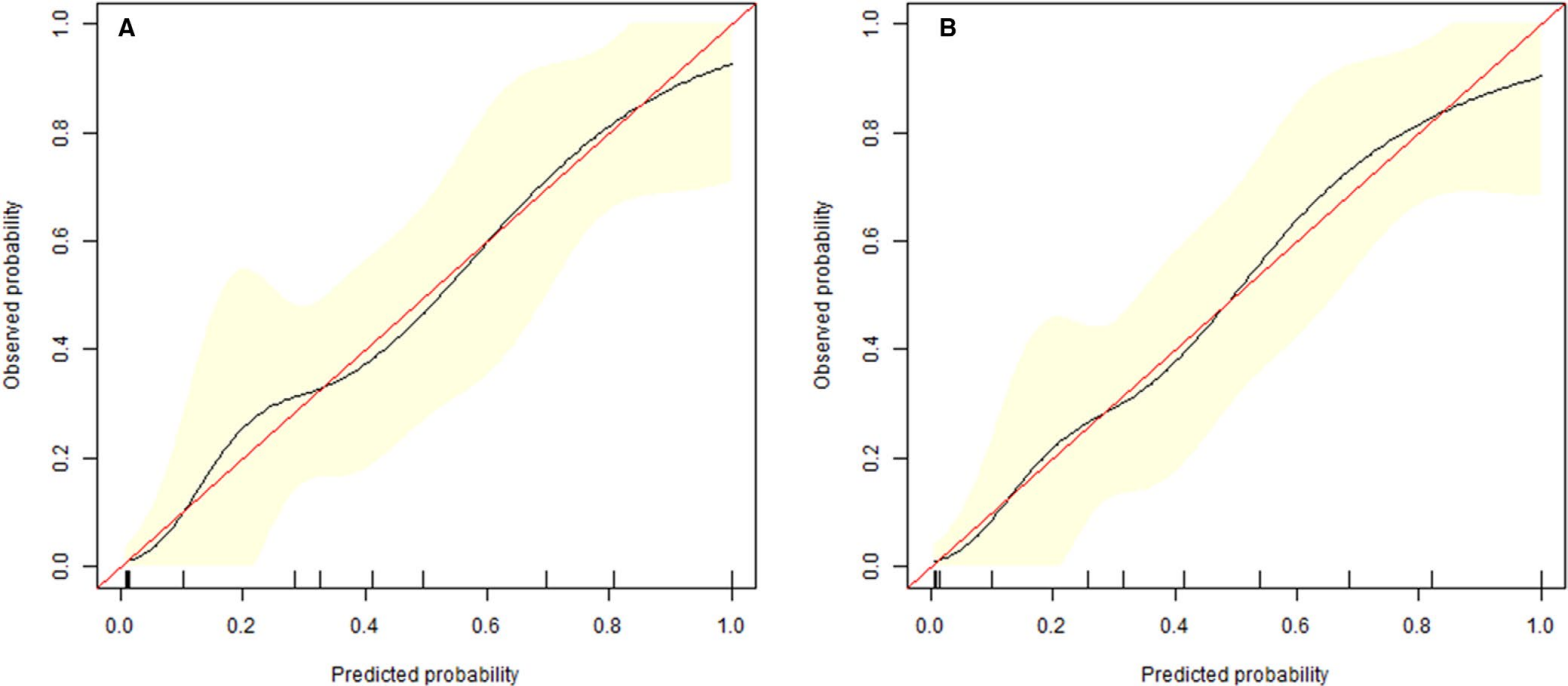
Calibration curve. Training set (A) and validation set (B)

A univariate logistic regression analysis identified the radiomics signature (odds ratio, OR = 54.776), CEA (OR = 12.629), and CA19‐9 (OR = 10.000) as independent predictors of SLM in RC patients (Table 2). A predictive radiomics nomogram was constructed based on the multivariable logistical regression combined with the selected risk factors (CEA, OR = 8.040; CA19‐9, OR = 4.560) and radiomics signature (OR = 70.629) to develop a prediction model for SLM (Table 2; Figure 3). The favorable calibration of the radiomics nomogram showed a nonsignificant Hosmer‐Lemeshow test statistic in both the training and validation sets (*P* = .636, *P* = .731). The calibration curve was satisfactory in both sets (Figure 4).

**TABLE 2:** Logistic regression analyses of predicting synchronous liver metastasis

Variables	Univariate logistic regression		Multivariate logistic regression	
	OR (95% CI)	*P*	OR (95% CI)	*P*
Gender (male/female)	1.647 (0.589‐4.609)	.342	NA	NA
Age (y)	0.981 (0.936‐1.027)	.410	NA	NA
Location (lower/middle/upper)	0.749 (0.353‐1.588)	.451	NA	NA
mr T stage (T1‐2/T3‐4)	2.744 (0.833‐9.039)	.097	NA	NA
mr N stage (N0/N1‐2)	2.068 (0.927‐4.612)	.076	NA	NA
MRF (negative/positive)	2.100 (0.662‐6.663)	.208	NA	NA
EMVI (negative/positive)	2.474 (0.92‐6.665)	.073	NA	NA
CEA (negative/positive)	12.629 (3.964‐40.232)	<.0001	8.040 (2.043‐31.640)	.003
CA19‐9 (negative/positive)	10.000 (3.059‐32.687)	<.0001	4.560 (1.038‐20.041)	.045
Radiomics signature	54.776 (5.274‐568.922)	.0008	70.629 (3.969‐1256.803)	.004

Abbreviations: NA, not available; OR, odds ratio.

**FIGURE 5:**
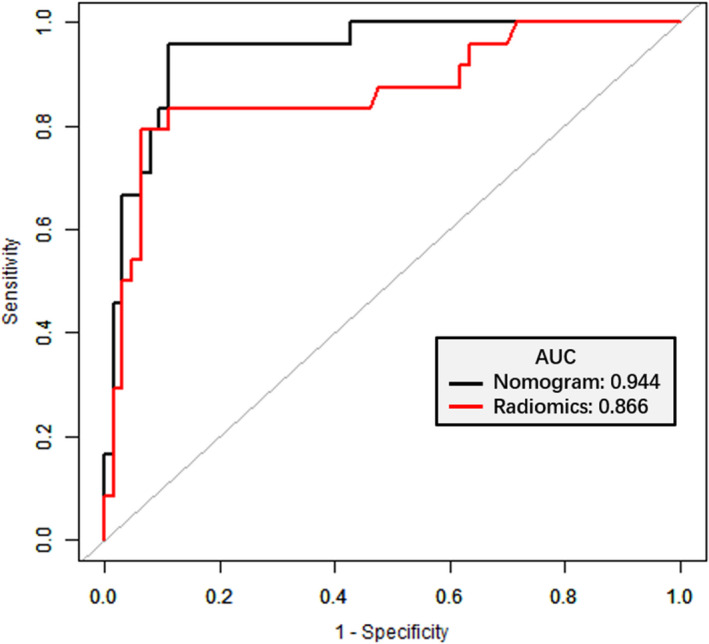
Receiver operator characteristic (ROC) curves (validation set). The prediction performance of the ROC curves for radiomics signature and radiomics nomogram for the validation set

The radiomics nomogram presented an accuracy of 81.58% for predicting SLM in the training set and 90.80% in the validation set. The AUC of radiomics nomogram was 0.918 (95% CI: 0.824‐1.000), the sensitivity was 90.00%, and the specificity was 78.57% in the training set, and that in the validation set was 0.944 (95% CI: 0.895‐0.993), 95.83%, and 88.90%, respectively (Table 3); simultaneously the radiomics model demonstrated an AUC of 0.866 (*P* = .044). In order to compare the prediction performance between the two models, the ROC curves were plotted for radiomics signature and radiomics nomogram for the validation set (Figure 5).

**TABLE 3:** Receiver operator characteristic analysis of the prediction model for the training and validation sets

	Training set		Validation set	
	Radiomics	Nomogram	Radiomics	Nomogram
AUC	0.836	0.918	0.866	0.944
95% CI	0.706‐0.965	0.824‐1.000	0.770‐0.963	0.895‐0.993
Sensitivity	100.0%	90.00%	79.17%	95.83%
Specificity	75.00%	78.57%	93.65%	88.89%
Accuracy	81.58%	81.58%	89.66%	90.80%
PLR	4.000	4.200	12.469	8.625
NLR	0.000	0.127	0.222	0.047
PPV	0.588	0.600	0.826	0.767
NPV	1.000	0.956	0.922	0.982
*P* *	0.170		0.044	

Abbreviations: NLR, negative likelihood ratio; NPV, negative predictive value; PLR, positive likelihood ratio; PPV, positive predictive value.

* Compared by DeLong test.

### 3.4 Clinical use

**FIGURE 6:**
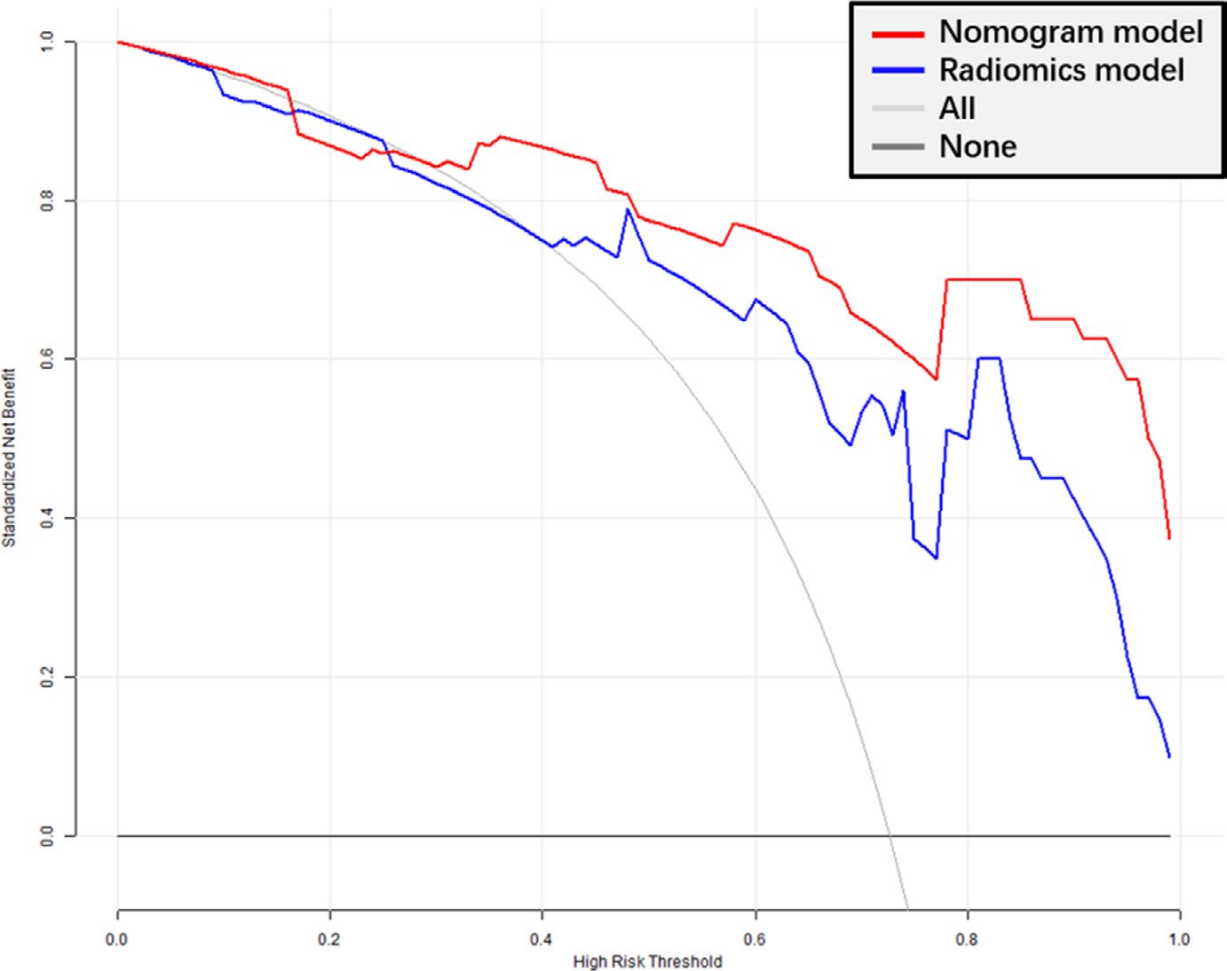
Decision curve analysis of the prediction model. *Y*‐axis represents the net benefit, which is calculated by gaining true positives and deleting false positives. The *X*‐axis is the probability threshold. The decision curve shows that if the probability of synchronous liver metastasis (SLM) ranges from 30% to 100%, using the radiomics nomogram to predict SLM provides more net benefit

The DCA (Figure 6) showed an adequate performance for radiomics and nomogram models in predicting SLM. When the threshold probability was within 0.3‐1.0, the proposed nomogram model to predict SLM showed a greater advantage than either the “all” or “none” scheme.

## 4 DISCUSSION

In this study, our results demonstrated that the radiomics nomogram provided predictive information for SLM in the primary RC. Contrast‐enhanced CT, MRI, and PET‐CT are common imaging examinations for the diagnisis of SLM in RC preoperatively. However, the sensitivity and accuracy of these imaging techniques are not satisfactory.^5^, ^6^, ^7^ According to one meta‐analysis, the detection sensitivity of colorectal LM in contrast‐enhanced CT, routine MRI, and FDG PET‐CT were 63%‐80%, 76%‐85.7%, and 51%‐90%, respectively.^23^ Therefore, screening for high‐risk predictors would improve the probability of early detection of SLM in RC patients. Typically, the clinicopathological predictors of SLM in RC patients include the histological type, pathological grade, depth of tumor invasion, lymph node status, vascular invasion, and tumor markers.^24^ However, some of these predictors can only be obtained postoperatively, and hence, inappropriate to guide preoperative treatment. Other studies have demonstrated that some features of rectal MRI, such as extramural vascular invasion, higher T stage, and regional lymph node metastasis are potential predictors.^25^, ^26^ However, these image features are subjective and qualitative, lacking quantitative assessment.

Recently, radiomics has been considered as an advanced tool to evaluate the tumor heterogeneity with respect to tumor diagnosis and prognosis prediction. Several studies have shown that radiomics model derived from primary tumors demonstrated an optimal performance in the prediction of SLM in RC patients.^19^, ^22^ Therefore, a radiomics nomogram can be obtained preoperatively based on T2WI and is widely accepted as the critical sequence for the preoperative assessment of primary tumors of RC.

In the current study, the factors including radiomics signature, CEA, and CA19‐9 levels were entered in the multivariate logistic regression to construct a predictive model and nomogram. The results were partially consistent with those from other relevant studies.^8^, ^19^, ^22^ Furthermore, the MR T stage, MR N stage, MRF, and EMVI were not independent image predictors for SLM according to the univariate analysis, which differed partially from that shown in previous studies describing the high risk of distant metastasis.^25^, ^26^ This phenomenon could be attributed to some nonquantitative features found on rectal MRI, which might underlie the lack of the clinical significance.

Therefore, our analysis indicates that the radiomics nomogram combined with tumor markers was superior to the radiomics signature alone. It exhibited a high predictive performance for SLM in RC patients, and the AUC improved from 0.866 to 0.944; the sensitivity was high in the validation cohort compared to radiomics model. Moreover, the results were better than those reported in a previous study on a per‐patient basis, wherein the AUC was 0.92 and 0.88 (MRI readers), 0.80 and 0.82 (CT readers), and with an AUC of 0.83 and 0.84 (PET‐CT readers).^27^

The detection sensitivity of SLM was 95.83% in the validation set, which was higher than the hepatobiliary phase MRI with a sensitivity of 90.6% for detecting LM in a meta‐analysis.^28^ The study also revealed an excellent sensitivity (95.5%) of gadoxetic acid‐enhanced MRI combined with diffusion‐weighted imaging (DWI) in detecting LM was. However, these findings were based on hepatocyte‐specific‐enhanced MRI, complicated and controversial procedure.

The NLR (negative likelihood ratio) was 0.047 in the validation set, as calculated by dividing the false‐negative rate by the true‐negative rate. An NLR < 0.10 excludes the disease. Therefore, the current data reflect the ability of the radiomics nomogram to exclude non‐SLM. Presuming that a patient is predicted to be non‐SLM by the radiomics nomogram, he/she may not benefit from radical liver imaging.

In this study, we constructed a radiomics nomogram to predict SLM based on primary RC from the rectal high‐resolution T2WI, which was widely used for assessing RC. Driven from the clinical risk factors and radiomics features, the proposed nomogram could be a valuable prediction tool for SLM in patients with RC. It can be utilized easily for identifying patients requiring further liver imaging.

Nevertheless, the present research has several limitations. First, with the relatively small sample size based on single institutional retrospective analysis, selection bias is inevitable. Second, the current study lacks external validation, and thus, large multicenter trials are needed to improve the universality of results. Finally, we only used T2WI‐based radiomics features to build the radiomics nomogram; other routine sequences, such as DWI, were not included in the present study. Further studies should be conducted using DWI, which might improve the predictive value of radiomics nomogram.

## 5 CONCLUSIONS

In conclusion, we developed a clinical‐radiomics nomogram by combining the tumor markers with radiomics signature to predict the presence of SLM in RC patients accurately. This visualization tool would detect the probability of SLM and aid the physician in clinical decisions.

## 6 FUNDING INFROMATION

The study was supported by the National Key Clinical Specialist Construction Programs of China (grant no.: N/A) and Youth Initiative Fund of Naval Medical University (grant no.: 2018QN05).

## CONFLICT OF INTEREST

The authors declare that they have no competing interests.

## AUTHORS' CONTRIBUTIONS

JL and FS conceived of the present idea. YX and YJ analyzed and interpreted the patient data regarding the radiomics features. XM and ML was a major contributor in writing the manuscript. All authors read and approved the final manuscript.

## Data Availability

The datasets used and/or analyzed during the current study are available from the corresponding author on reasonable request.
